# Non-Covalent Loading of Anti-Cancer Doxorubicin by Modularizable Peptide Self-Assemblies for a Nanoscale Drug Carrier

**DOI:** 10.3390/molecules22111916

**Published:** 2017-11-06

**Authors:** Kin-ya Tomizaki, Kohei Kishioka, Shunsuke Kataoka, Makoto Miyatani, Takuya Ikeda, Mami Komada, Takahito Imai, Kenji Usui

**Affiliations:** 1 Department of Materials Chemistry, Ryukoku University, 1-5 Yokotani, Seta-Oe, Otsu 520-2194, Japan; k-kishioka@daitochemix.co.jp (K.K.); t16m055@mail.ryukoku.ac.jp (S.K.); eengaww.7762@gmail.com (M.M.); ti19900114@icloud.com (T.I.); imai@rins.ryukoku.ac.jp (T.I.); 2 Innovative Materials and Processing Research Center, Ryukoku University, 1-5 Yokotani, Seta-Oe, Otsu 520-2194, Japan; 3 Faculty of Frontiers of Innovative Research in Science and Technology (FIRST), Konan University, 7-1-20 Minatojima-Minami, Chuo, Kobe 650-0047, Japan; mkmkmk991012@gmail.com

**Keywords:** peptide self-assembly, drug delivery system, cell-penetrating peptide, nuclear-localization signal, anti-cancer doxorubicin

## Abstract

We prepared nanoscale, modularizable, self-assembled peptide nanoarchitectures with diameters less of than 20 nm by combining β-sheet-forming peptides tethering a cell-penetrating peptide or a nuclear localization signal sequence. We also found that doxorubicin (Dox), an anti-cancer drug, was non-covalently accommodated by the assemblies at a ratio of one Dox molecule per ten peptides. The Dox-loaded peptide assemblies facilitated cellular uptake and subsequent nuclear localization in HeLa cells, and induced cell death even at low Dox concentrations. This peptide nanocarrier motif is a promising platform for a biocompatible drug delivery system by altering the targeting head groups of the carrier peptides.

Targeted drug delivery systems, especially those targeted to the nucleus, have drawn great attention because they have the promise to depress toxic side effects and simultaneously enhance therapeutic efficiency. For instance, gene therapy involves correcting dysfunctional and missing genes by delivering therapeutic genes into the cell nucleus [1]. The nuclear envelope contains unique channels called nuclear pore complexes (NPCs), which allow passive diffusion of ions and small molecules (<40 kDa) through aqueous channels with a diameter of 20–70 nm [2,3]. Molecules larger than 25 nm in diameter can be transported across the NPCs by importin α/β-mediated nuclear import and export systems [4]. Thus, the intranuclear delivery of relatively large particles, such as drug carriers can be achieved with the assistance of nuclear localization signals (NLSs) for passing through NPCs [5,6,7]. The NLSs of the viral proteins or protein transduction domains consist of relatively short peptides (<30 amino acids) and include HIV-1 Tat (48–60) [8], HIV-1 Rev (34–50) [9], and SV40 T-antigen (126–132) [10]. The highly effective eight-arginine-sequence [(Arg)_8_] translocates large molecules into cells and belongs to a class known as cell-penetrating peptides (CPPs) [9,11]. So far, covalent linkages or simple physical interactions of CPPs have been demonstrated to promote the cellular uptake of a variety of particles, such as DNA [12], peptides [13], proteins [14], liposomes and micelles [15], and quantum dots [16]. A small anticancer drug, doxorubicin (Dox), was previously delivered by conjugation with a carrier peptide to the mitochondria [17]. Diversifying the platform of drug carriers having diameters less than NPCs and/or requiring no covalent linkages with small drugs is an attractive method to improve the targeted drug delivery [18,19,20,21,22].

We have so far devoted our efforts to the design, synthesis, and characterization of self-assembled peptide nanoarchitectures, and have found that RU006, a β-sheet-forming nonapeptide, forms a disk-like assembly that is approximately 50 nm wide and 2.5 nm thick [23,24], which is comparable to NPCs. For intracellular targeted drug delivery, if CPP/NLS-modified RU006 peptides can be used, the modularized assembly of the peptides would be effective for nuclear localization of drugs via not only passive diffusion, but also through endosomal escape and NPC transfer systems. Furthermore, small drugs can be accommodated into the particles during self-assembly without covalent linkages that might alter their biological effects (Figure 1). Here, we describe (i) the design and synthesis of CPP/NLS-modified RU006 peptides, (ii) the characterization and cellular uptake/nuclear localization of the modularized peptide assemblies, and (iii) non-covalent loading of Dox by the assemblies and Dox-induced cell death activity. This is a potentially versatile and modularizable strategy for the development of targeted drug delivery systems.

The design of the amphiphilic nonapeptide RU006 was described previously [23,24]. Briefly, RU006 was designed to have two isoleucines and a 2-naphthylalanine at the hydrophobic face, which provides the driving force for self-assembly via hydrophobic interactions and π-π stacking (Figure 2A). Four alanines and a lysine (Lys) were placed at the hydrophilic face to make the peptide water-soluble. Another Lys residue was arranged at the hydrophobic face to make it favorable for packaging the organic molecules via hydrogen bonding and/or electrostatic interactions. The sequences of SV40 T-antigen (126–132) [10], (Arg)_8_ [9,11], and HIV-1 Tat (47–60) [8] were chosen to design NLS-RU006, R8-RU006, and Tat-RU006 as CPP/NLS-tethering peptides, respectively. The N-terminus of NLS-RU006 was modified with biotin to determine the arrangement of the NLS moieties in the carrier architectures. As an intracellular tracer, 5(6)-carboxytetramethylrhodamine (TMR)-capped RU006 was also designed (TMR-RU006). These head groups were attached at the N-terminus of the RU006 sequence via two or three-glycine-repeats. The peptides were synthesized by the standard solid-phase peptide synthetic method [25], purified by reverse-phase HPLC, and were characterized by MALDI-TOF MS (Figures S1–S8).

Two sets of four different RU006-based peptide nanocarriers were prepared (Figure 2B): Entry 1 involving NLS-RU006, R8-RU006, and Tat-RU006; Entries 2, 3 and 4 lacking two of the three CPP/NLS-peptides; and Entries 1’–4’ including TMR-RU006 in addition to Entries 1–4 for the detection of peptide localization in cells. Total peptide concentrations for self-assembly were kept constant at 100 μM, and to lower cytotoxicity and to avoid the destabilization of the peptide self-assemblies, RU006 was mixed with its derivative(s) tethering strongly-basic and bulky head group(s) in 80/20 (79/21) ratio(s). They formed nanocarriers in 20 mM sodium phosphate buffer, 150 mM NaCl (pH 7.2) at 25 °C.

First, we attempted to characterize the peptide self-assemblies of Entry 1 (100 μM in 20 mM sodium phosphate buffer, 150 mM NaCl, pH 7.2) by dynamic light scattering (DLS). Unfortunately, DLS signals corresponding to the assemblies were not obtained reproducibly, probably due to the relatively diluted concentration of the peptide. Thus, we characterized the morphology of the peptide nanocarriers of Entry 1 by transmission electron microscopy (TEM). Figure S9 and Figure 3A,B show the presence of round-shaped assemblies with diameters of 9 ± 3 nm, 12 ± 5 nm, 14 ± 6 nm, and 14 ± 6 nm, in width at 1, 6, 12 and 24 h after self-assembly started in buffer solution at 25 °C, respectively, which are comparable to the pore sizes of NPCs. However, aggregation of the nanoscale assemblies was observed after a 12-h incubation. Atomic force microscopic (AFM) measurements revealed disk-like assemblies of approximately 2 nm thick, as seen in the literature [18,19] by a 6-h incubation (Figure 3C).

Thereafter, we decided to use a 6-h incubation for self-assembly. Treatment of peptide nanocarriers of Entry 1 with gold colloidal particle (10 nm)-labeled goat anti-biotin antibody on the TEM grid resulted in gold nanoparticles (black spheres) on the gray-colored assemblies, indicating that NLS moieties were expectedly sticking out from the surface of the assemblies into the aqueous media (Figure 3D). Other peptide nanocarriers of Entries 2, 3 and 4 also showed round-shaped assemblies of 14 ± 8 nm, 13 ± 6 nm and 13 ± 6 nm in width at 6 h, respectively (Figure S10). With these self-assembled peptide nanocarriers in hand, we performed cellular uptake assays.

To investigate the cellular uptake of peptide nanocarriers of Entries 1’–4’, we mixed peptide-containing solution matured by a 6-h pre-incubation (100 μM) and cell culture medium in equivolume ratios, incubated HeLa cells with peptides at a final concentration of 50 µM for 4 h at 37 °C in a 5% CO_2_ atmosphere, and examined the cellular uptake and subsequent localization of the peptide nanocarriers in the cells after a 4-h incubation (Figure 4). Confocal laser scanning microscopy (CLSM) imaging revealed that the red fluorescence signals for TMR-RU006 were superimposed with blue ones for Hoechst33342 emission, as shown in Figure 4B, likely indicating that peptide nanocarriers of Entry 2’ successfully localized in the HeLa nuclei without cytotoxicity at concentrations ranging from 1 to 100 μM (Figure S11A). Unfortunately, the passive diffusion process that we expected appears to have been minor, because peptide nanocarriers of Entries 1’, 3’ and 4’ showed the limited delivery of TMR-RU006 molecules into the nuclei, but were still trapped in the cytoplasm and the perinuclear region without cytotoxicity even after a 4-h incubation (Figure 4A,C,D and Figure S11B) [26]. Seymour et al. reported that the NLS sequence from SV40 T-antigen (PKKKRKVEDPYC) significantly binds to importin α1, which is an intracellular transport receptor, and facilitates the transfer through nuclear pores [27]. Therefore, these results suggest that NLS-RU006 accompanied by TMR-RU006 was delivered into HeLa nuclei via the nuclear import pathway in an NLS-concentration dependent fashion.

Next, we attempted to examine Dox encapsulation by RU006 carrier peptides and estimate the amounts of Dox loaded into the assemblies by gel filtration chromatography. However, peaks for peptide self-assemblies were not observed, but rather we observed that of the peptide monomer (data not shown). The peak for Dox incubated with RU006 was detected a little earlier than that for Dox only, indicating that Dox and the peptide formed apparently larger-molecular-weight constructs (Figure S12). These results suggest that even though the peptide and Dox associated weakly and was dissociated by interactions in the gel packed column during measurements, Dox might be encapsulated within the peptide self-assemblies. Therefore, we examined the Dox encapsulation process by fluorescence labeling. We first confirmed a linear correlation between Dox concentration and fluorescence intensity in the dynamic range for this study (Figure S13). Figure 5A shows the fluorescence emission from Dox before (10 μM) and after ultrafiltration with a cutoff molecular weight (COMW) of 100 kDa. The filtrates of the buffer solution containing Dox without RU006 showed fluorescence intensities that decreased to approximately 5% relative to that before ultrafiltration (control), suggesting that Dox molecules were favorably adsorbed onto the ultrafiltration membrane. With increasing RU006 concentration to 100 μM, Dox fluorescence of the filtrates increased up to 80% relative to the control.

Even when the pore size of the ultrafiltration membrane was decreased from 100 kDa to 3 kDa COMW, approximately 60% of Dox fluorescence relative to the control was still observed in the filtrates (Figure S14). Furthermore, we estimated the amounts of peptides that passed through ultrafiltration membranes by HPLC. Figure S15 shows HPLC profiles for RU006 peptide nanocarriers before (100 μM) and after ultrafiltration, resulting in an approximate 25% decrease in HPLC signal intensity by ultrafiltration. Dox peaks were too small to detect by the HPLC measurement due to the small extinction coefficient for Dox. These ultrafiltration and HPLC data indicated that Dox molecules were successfully accommodated into peptide nanocarriers, favorably forming nanoparticles with sizes of less than 10–100 kDa, and further allowed us to infer that the assemblies accommodated Dox at one Dox molecule per ten peptides (maximum one Dox per five peptides) with an approximate 80% efficiency.

We also performed Dox release experiments from Dox-loaded RU006 nanocarriers (Figure S16). The buffer solution containing Dox-loaded RU006 nanocarriers after a 6-h incubation for self-assembly/encapsulation was mixed with cell culture medium in an equivolume ratio, and incubated at 37 °C for 0, 1, 2, 3, and 4 h. At these time points, sample solutions were filtered through an ultrafiltration membrane filter (COMW 100 kDa) to remove free Dox molecules. The results showed that Dox molecules were stably encapsulated in the peptide nanocarriers and were released slowly from the carriers at a rate of 10% release per 4 h, sufficient for cellular uptake. 

Last, we conducted Dox-induced cell death assays for HeLa cells by incubation with an equivolume mixture of buffer solution containing peptide nanocarriers of Entry 2 (6-h pre-incubation sample for maturation) that showed excellent nuclear localization potential (Figure 4B) and cell culture medium (final Dox and peptide concentrations = 5.0 and 50 μM, respectively) for 24 h at 37 °C in a 5% CO_2_ atmosphere [26]. Figure 5B shows the cytotoxicity of the solutions of Dox only, Dox + RU006, and Dox + Entry 2, indicating that cell viability gradually decreased with increasing Dox concentration from 0.1 to 5.0 μM. Notably, the use of peptide nanocarriers of Entry 2 effectively induced cell death even at low Dox concentrations (~0.1 μM), indicating that NLS-tethering self-assembled peptide nanocarriers successfully delivered small drugs to HeLa nuclei without the need for covalent linkages. Meanwhile, no significant difference in cell viability between Dox-only and Dox + peptide carriers at relatively high 5.0 μM of Dox concentration was observed. This is possibly due to the encapsulation of some of Dox molecules within relatively large aggregates that were ineffective for cellular uptake, but the details are still unclear.

In conclusion, we have examined self-assembled peptide nanoarchitectures for nuclear-targeted drug delivery. Incorporating the CPP/NLS-moieties, including SV40 T-antigen (126–132), (Arg)_8_, and HIV-1 Tat (47–60) sequences, into the peptide assemblies and improved cellular uptake and subsequent nuclear localization by assistance of the NLS moiety. The assemblies also accommodated small drugs such as Dox inside of them without covalent linkages that would probably alter the biological effects of the drugs. This peptide nanocarrier system can serve as a versatile, modularizable platform for targeted drug delivery by altering the targeting head groups of the carrier peptides for use against cancer cells of interest (e.g., breast cancer and ovarian cancer). Further characterization of the colloidal properties of the peptide nanocarriers is needed. This will include fluorescence correlation microscopy, estimating the stability of the assemblies in the presence of serum, and elucidating the mechanisms of cellular uptake and nuclear localization. Future work will also apply the system to different cancer cells using different drugs to maximize its potential for drug delivery.

## Figures and Tables

**Figure 1:**
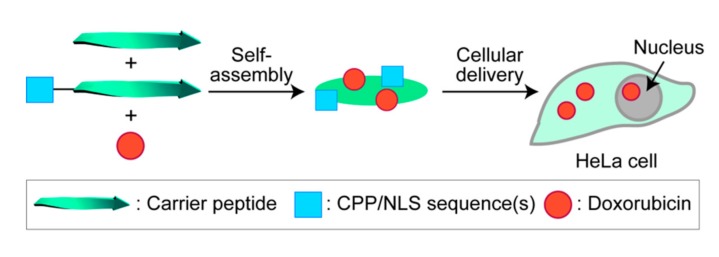
Schematic illustration of intracellular delivery of doxorubicin by modularized peptide assemblies as nanocarriers.

**Figure 2:**
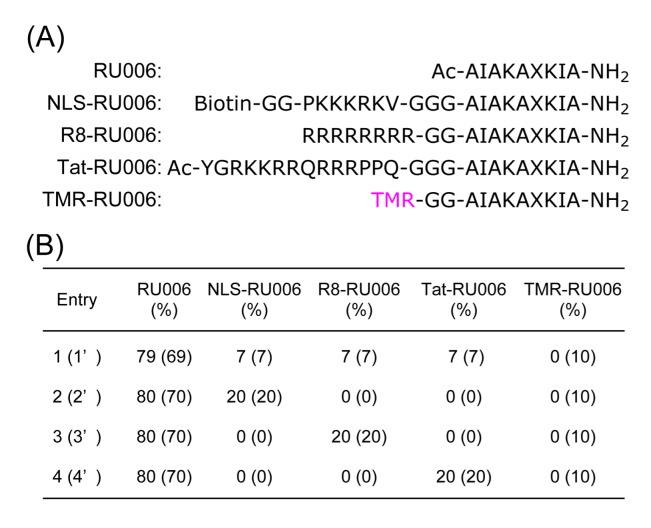
(**A**) Amino acid sequences of RU006 as a carrier peptide; NLS-RU006, R8-RU006, and Tat-RU006 as CPP/NLS moiety-tethering peptides; and TMR-RU006 as a fluorescence tracer; (**B**) The compositions of nanocarriers examined in this study. The compositions of Entries 1’–4’ are in parentheses. Abbreviations: CPP = cell-penetrating peptide; NLS = nuclear localization signal; X = L-2-naphthylalanine; TMR = 5(6)-carboxytetramethylrhodamine.

**Figure 3:**
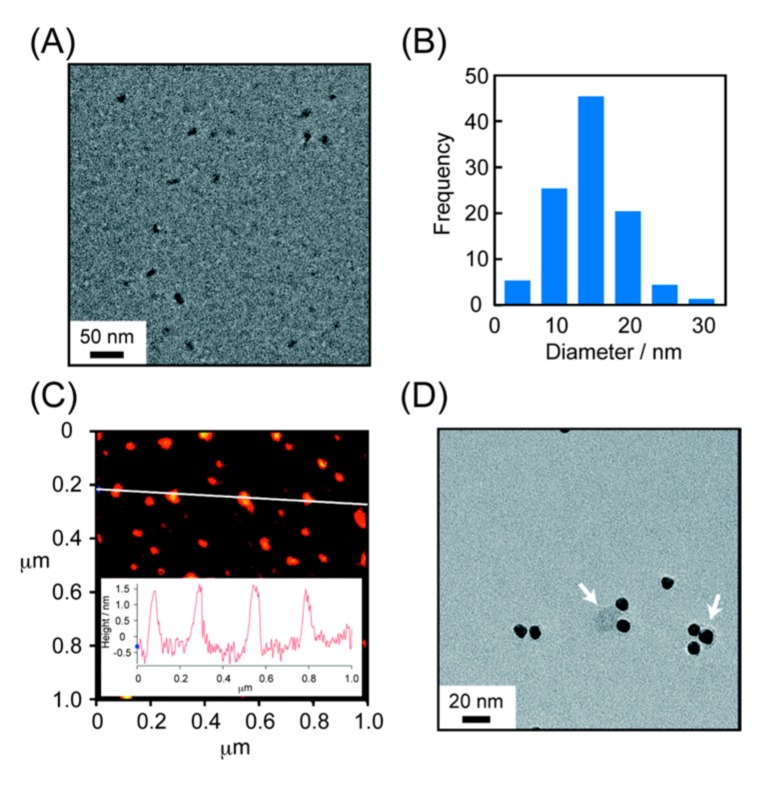
Characterization of self-assembled peptide nanocarriers of Entry 1 prepared by incubation at 25 °C for 6 h ([peptide] = 100 μM in 20 mM sodium phosphate buffer, 150 mM NaCl, pH 7.2). (**A**) Transmission electron microscopy (TEM) image of the self-assembled peptide nanocarriers; (**B**) distribution in diameters of the nanocarriers in TEM images; (**C**) atomic force microscopic (AFM) analysis of the nanocarriers; and (**D**) localization of the NLS-moieties of the nanocarriers by immunostaining with gold colloidal particle (10 nm)-labeled goat anti-biotin antibody. Black spheres show gold nanoparticles and white arrows indicate self-assembled peptide nanocarriers colored in gray in panel three-dimensional (3D). TEM samples were negatively stained with 2% phosphotungstic acid.

**Figure 4:**
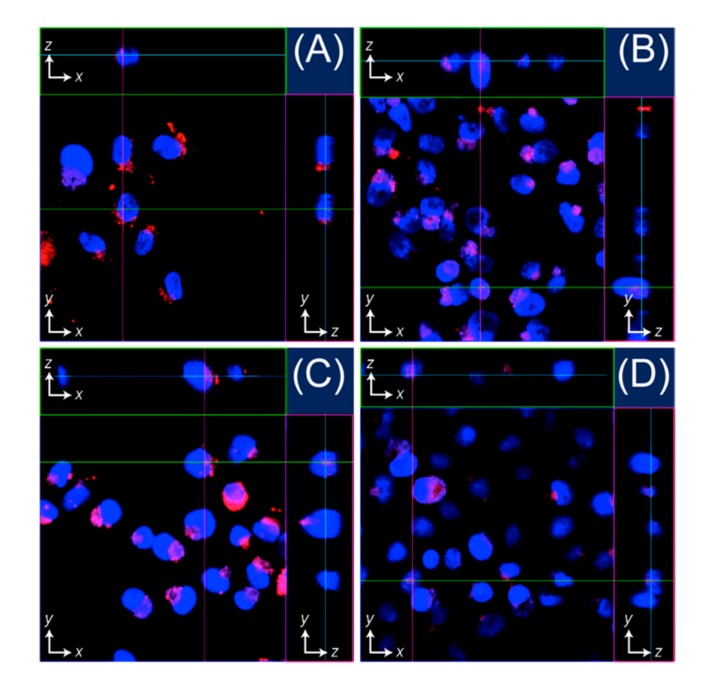
Confocal laser scanning microscopy images (40×) of HeLa cells after incubation with (**A**) Entry 1’, (**B**) Entry 2’, (**C**) Entry 3’, and (**D**) Entry 4’ at 37 °C in a 5% CO_2_ atmosphere for 4 h ([peptide] = 50 μM). Red and blue signals indicate fluorescence emissions from 5(6)-carboxytetramethylrhodamine (TMR) and Hoechst 33342, respectively.

**Figure 5:**
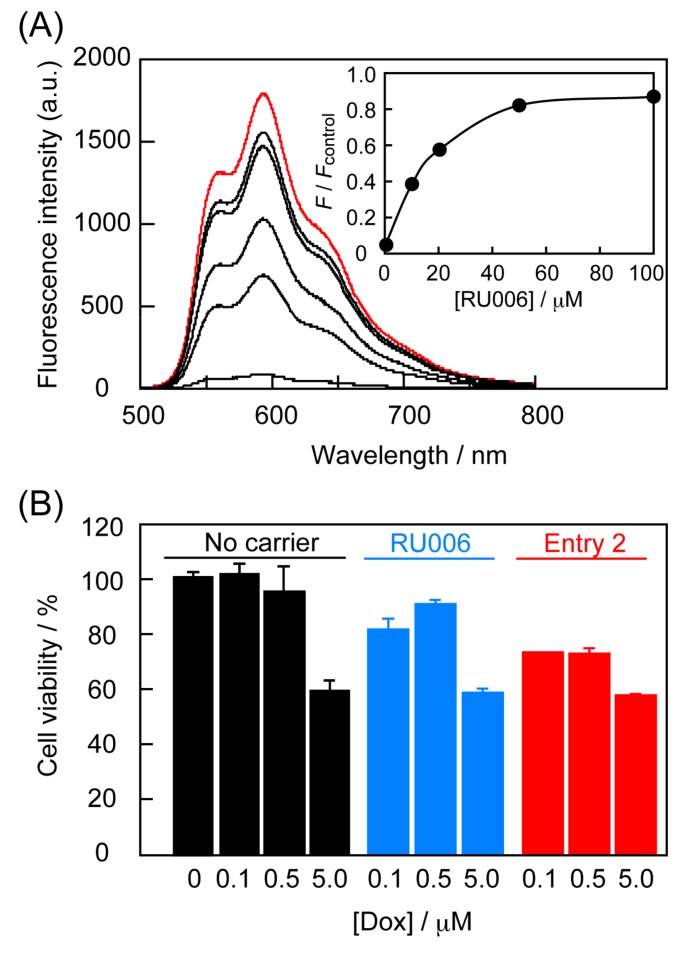
(**A**) Doxorubicin (Dox) encapsulation by RU006 carrier peptides as a function of RU006 concentration ([Dox] = 10 μM and [RU006] = 0, 10, 20, 50, and 100 μM in 20 mM sodium phosphate buffer, 150 mM NaCl, pH 7.2 at 25 °C for 6 h) detected by fluorescence intensity from Dox (λ_ex_ = 480 nm) after ultrafiltration (COMW 100 kDa). Red line shows fluorescence emission from Dox without RU006 before ultrafiltration as a control. Inset shows relative fluorescence intensity of Dox-loaded RU006 peptide nanocarriers at 594 nm. *F*_control_ indicates Dox fluorescence intensity for the red line in panel 5A; (**B**) Cytotoxicity (Dox-induced cell death) of Dox-loaded self-assembled peptide nanocarriers of Entry 2 for HeLa cells at 24 h after the reaction started (final peptide concentration = 50 μM) (mean ± SD, n ≥ 3). Control experiments involved cell culture lacking both Dox and peptides.

## Supplementary Materials

Supplementary Material is available online. Materials and Methods, characterization data (HPLC and MALDI-TOF MS profiles) for the peptides, characterization of the self-assembled peptide nanocarriers of Entries 1, 2, 3 and 4, cytotoxicity of self-assembled peptide nanocarriers for HeLa cells, doxorubicin encapsulation by RU006 peptide nanocarrier characterized by ultrafiltration assay, doxorubicin encapsulation by RU006 peptide nanocarrier characterized by gel filtration chromatography, HPLC and fluorescence spectroscopy, and references.

## References

[1] Van der Aa M.A., Mastrobattista E., Oosting R.S., Hennink W.E., Koning G.A., Grommelin D.J.A. (2006). The nuclear pore complex: The gateway to successful nonviral gene delivery. Pharm. Res..

[2] Kubitscheck U., Grünwald D., Hoekstra A., Rohleder D., Kues T., Siebrasse J.P., Peters R.J. (2005). Nuclear transport of single molecules: Dwell times at the nuclear pore complex. Cell Biol..

[3] Misra R., Sahoo S.K. (2010). Intracellular trafficking of nuclear localization signal conjugated nanoparticles for cancer therapy. Eur. J. Pharm. Sci..

[4] Lange A., Mills R.E., Lange C.J., Stewart M., Devine S.E., Corbett A.H. (2007). Classical nuclear localization signals: Definition, function, and interaction with importin alpha. J. Biol. Chem..

[5] Nakielny S., Dreyfuss G. (1999). Transport of proteins and RNAs in and out of the nucleus. Cell.

[6] Patel S.S., Belmont B.J., Sante J.M., Rexach M.F. (2007). Natively unfolded nucleoporins gate protein diffusion across the nuclear pore complex. Cell.

[7] Alber F., Dokudovskaya S., Veenhoff L.M., Zhang W., Kipper J., Devos D., Suprapto A., Karni-Schmidt O., Williams R., Chait B.T. (2007). The molecular architecture of the nuclear pore complex. Nature.

[8] Vivès E., Brodin P., Lebleu B. (1997). A truncated HIV-1 Tat protein basic domain rapidly translocates through the plasma membrane and accumulates in the cell nucleus. J. Biol. Chem..

[9] Futaki S., Suzuki T., Ohashi W., Yagami T., Tanaka S., Ueda K., Sugiura Y. (2001). Arginine-rich peptides. An abundant source of membrane-permeable peptides having potential as carriers for intracellular protein delivery. J. Biol. Chem..

[10] Qu W., Qin S.-Y., Ren S., Jiang X.-J., Zhou R.-X., Zhang X.-Z. (2013). Peptide-based vector of VEGF plasmid for efficient gene delivery in vitro and vessel formation in vivo. Bioconjugate Chem..

[11] Nagel Y.A., Raschle P.S., Wennemers H. (2017). Effect of preorganized charge-display on the cell-penetrating properties of cationic peptides. Angew. Chem. Int. Ed..

[12] Veldhoen S., Laufer S.D., Restle T. (2008). Recent developments in peptide-based nucleic acid delivery. Int. J. Mol. Sci..

[13] Dostmann W.R., Taylor M.S., Nickl C.K., Brayden J.E., Frank R., Tegge W.J. (2000). Highly specific, membrane-permeant peptide blockers of cGMP-dependent protein kinase Ialpha inhibit NO-induced cerebral dilation. Proc. Natl. Acad. Sci. USA.

[14] Säälik P., Elmquist A., Hansen M., Padari K., Saar K., Viht K., Langel &#xDC, Pooga M. (2004). Protein cargo delivery properties of cell-penetrating peptides. A comparative study. Bioconjugate Chem..

[15] Torchilin V.P. (2008). Tat peptide-mediated intracellular delivery of pharmaceutical nanocarriers. Adv. Drug Deliv. Rev..

[16] Medintz L., Pons T., Delehanty J.B., Susumu K., Brunel F.M., Dawson P.E., Mattoussi H. (2008). Intracellular delivery of quantum dot-protein cargos mediated by cell penetrating peptides. Bioconj. Chem..

[17] Chamberlain G.R., Tulumello D.V., Kelley S.O. (2013). Targeted delivery of doxorubicin to mitochondria. ACS Chem. Biol..

[18] Raad M.D., Teunissen E.A., Mastrobattista E. (2014). Peptide vectors for gene delivery: From single peptides to multifunctional peptide nanocarriers. Nanomedicine.

[19] Sundar S., Chen Y., Tong Y.W. (2014). Delivery of therapeutics and molecules using self-assembled peptides. Curr. Med. Chem..

[20] Sharma P.P., Rathi B., Rodrigues J., Gorobets N.Y. (2015). Self-assembled peptide nanoarchitectures: Applications and future aspects. Curr. Top. Med. Chem..

[21] Habibi N., Kamaly N., Memic A., Shaflee H. (2016). Self-assembled nanstructures: Smart nanomaterials toward targeted drug delivery. Nano Today.

[22] Eskandari S., Guerin T., Toth I., Stephenson R.J. (2017). Recent advances in self-assembled peptides: Implications for targeted drug delivery and vaccine engineering. Adv. Drug Deliv. Rev..

[23] Tomizaki K.-Y., Wakizaka S., Yamaguchi Y., Kobayashi A., Imai T. (2014). Ultrathin gold nanoribbons synthesized within the interior cavity of a self-assembled peptide nanoarchitecture. Langmuir.

[24] Tomizaki K.-Y., Kishioka K., Kobayashi H., Kobayashi A., Yamada N., Kataoka S., Imai T., Kasuno M. (2015). Roles of aromatic side chains and template effects of the hydrophobic cavity of a self-assembled peptide nanoarchitecture for anisotropic growth of gold nanocrystals. Bioorg. Med. Chem..

[25] Chan W.C., White P.D. (2000). Fmoc Solid Phase Peptide Synthesis: A Practical Approach.

[26] Chen G., Xie Y., Peltier R., Lei H., Wang P., Chen J., Hu Y., Wang F., Yao X., Sun H. (2016). Peptide-decorated gold nanoparticles as functional nano-capping agent of mesoporous silica container for targeting drug delivery. ACS Appl. Mater. Interfaces.

[27] Bremner K.H., Seymour L.W., Logan A., Read M.L. (2004). Factors influencing the ability of nuclear localization sequence peptides to enhance nonviral gene delivery. Bioconjugate Chem..

