# Supplementary material for: Non-Covalent Loading of Anti-Cancer Doxorubicin by Modularizable Peptide Self-Assemblies for a Nanoscale Drug Carrier
Source: Molecules. 2017 Nov 6;22(11):1916. doi: 10.3390/molecules22111916 (PMC6150382; doi:10.3390/molecules22111916)
Supplement: Supplementary file 1 [file molecules-22-01916-s001.pdf]

Supplementary Material

for

Non-Covalent Loading of Anti-Cancer Doxorubicin by Modularizable Peptide Self-Assemblies for a Nanoscale Drug Carrier

Kin-ya Tomizaki,*[1,2] Kohei Kishioka,[1] Shunsuke Kataoka,[1] Makoto Miyatani,[1] Takuya Ikeda,[1] Mami Komada,[3] Takahito Imai,[1] and Kenji Usui*[3]

[1]*Department of Materials Chemistry and* [2]*Innovative Materials and Processing Research Center, Ryukoku University, 1-5 Yokotani, Seta-Oe, Otsu 520-2194, Japan.*
Fax: +81 77 543 7483; Tel: +81 77 543 7469; E-mail: tomizaki@rins.ryukoku.ac.jp
[3]*Faculty of Frontiers of Innovative Research in Science and Technology (FIRST), Konan University, 7-1-20 Minatojima-Minami, Chuo, Kobe 650-0047, Japan.*
Fax: +81 78 303 1495; Tel: +81 78 303 1418; E-mail: kusui@center.konan-u.ac.jp

CONTENTS

(1) Materials and Methods.
(2) Characterization data (HPLC and MALDI-TOFMS profiles) for the peptides.
(3) Characterization of the self-assembled peptide nanocarriers of Entry 1.
(4) Characterization of the self-assembled peptide nanocarriers of Entries 1, 2, 3 and 4.
(5) Cytotoxicity of self-assembled peptide nanocarriers for HeLa cells.
(6) Gel filtration chromatography of Dox only and Dox-loaded RU006 peptide nanocarrier.
(7) Correlation between fluorescence intensity and concentration of Dox.
(8) Doxorubicin encapsulation by RU006 peptide nanocarrier characterized by ultrafiltration assay.
(9) Doxorubicin encapsulation by RU006 peptide nanocarrier characterized by HPLC.
(10) Doxorubicin release from Dox-loaded RU006 peptide nanocarrier.
(11) References

(1) Materials and Methods

General. All solvents and reagents, unless otherwise noticed, were purchased from Wako Pure Chemical Industries (Japan) and used as received. Fmoc-amino acid derivatives and reagents for peptide synthesis were purchased from Watanabe Chemical Industries (Japan). Acetonitrile (HPLC grade) was purchased from Nacalai Tesque Inc. (Japan) and used for HPLC analysis and purification. Ultrapure water (water) was purchased from Wako Pure Chemical Industries (Japan). 5(6)-Carboxytetramethylrhodamine was purchased from Sigma. Gold colloidal particle (10 nm)-labeled goat anti-biotin antibody was purchased from Cosmo Bio (Japan). Doxorubicin hydrochloride (Dox) was purchased from Wako Pure Chemical Industries (Japan).

Peptide Synthesis. Peptide nanocarriers, RU006,[S1] NLS-RU006, R8-RU006, Tat-RU006, and TMR-RU006 were synthesized by means of Fmoc chemistry on Rink Amide resin with 2-(1*H*-benzotriazole-1-yl)-1,1,3,3-tetramethyluronium hexafluorophosphate (HBTU) and 1-hydroxybenzotriazole monohydrate (HOBt) as coupling reagents.[S2] Amino acid side chains of lysine (Lys), arginine (Arg), tyrosine (Tyr) and glutamine (Gln) were protected with *t*-butyloxycarbonyl (Boc), 2,2,4,6,7-pentamethyldihydrobenzofuran-5-sulfonyl (Pbf), *t*-butyl (*t*Bu) and trityl (Trt), respectively. Final deprotection and deresination of peptide-bound resins were performed by treatment with trifluoroacetic acid (TFA)/thioanisole/*m*-cresol (90/7.5/2.5, *v*/*v*/*v*) at room temperature for 60 min. The obtained crude peptides were purified by Hitachi LaChrom Elite HPLC system (Japan) using Cosmosil $5C_{18}$-AR-II packed columns (4.6 x 150 mm and 10 x 250 mm, Nacalai Tesque, Japan) with a linear gradient of acetonitrile/0.1% TFA at flow rates of 1.0 mL min^{-1} and 3.0 mL min^{-1} for analysis and preparative purification, respectively. The purified peptides were lyophilized and characterized by matrix-assisted laser desorption/ionization time-of-flight mass spectrometry (MALDI-TOFMS, Shimadzu AXIMA-CFR Plus, Japan).

Preparation of Self-Assembled Peptide Nanocarriers. Peptide stock solution was prepared by dissolving the purified peptide powder in 2,2,2-trifluoroethanol (TFE) to prevent self-assembly during storage. The concentrations of the stock solutions for RU006, NLS-RU006, R8-RU006 and Tat-RU006 were determined by absorption spectroscopy (Shimadzu UV-3100 spectrophotometer, Japan) using an extinction coefficient of 5,500 $M^{-1}cm^{-1}$ at around 275 nm for the 2-naphthylalanine residue [Nal(2)] in aqueous solution

containing 1% TFE (*v*/*v*).[S3] The concentration of the stock solution for TMR-RU006 was determined by absorption spectroscopy (Shimadzu UV-3100 spectrophotometer, Japan) using an extinction coefficient of 80,400 $M^{-1}cm^{-1}$ at around 550 nm for the TMR in water/acetonitrile (1/1, *v*/*v*) containing 1% TFE. The peptide stock solution in TFE was transferred into a microtube, dried with a N_2 gas stream, then dried *in vacuo* for 1 h. Solvent (20 mM phosphate buffer, 150 mM NaCl, pH 7.2) was added to the microtube to be 100 μM of the total peptide concentration and the obtained aqueous solution was sonicated with a Kaijo Sono Cleaner (CA-44800, Japan) at 50 °C for 2 min to allow peptides being in monomer/oligomer states, then filtered through a spin filter (Ultrafree-MC-VV, 0.1 μm, Millipore). The filtrate was incubated in a low temperature incubator (Fukushima FMU-0531, Japan) at 25 °C for 6 h for self-assembly. The obtained samples were served for microscopic measurements and cellular delivery assays.

Transmission Electron Microscopy (TEM). Droplets of the solution containing self-assembled peptide nanocarriers (10 μL) were applied to a TEM grid (Cu 200 mesh covered with a collodion membrane, Nisshin EM, Japan) for 1 min, and the sample was negatively stained with 2% phosphotungstic acid for 1 min, washed with water, and dried with a filter paper. All TEM samples were dried *in vacuo* before TEM measurements were conducted at an accelerating voltage of 200 kV (JEOL JEM-2100, Japan).

Separately, TEM samples for immunostaining was prepared as follows: Droplets of the solution containing self-assembled peptide nanocarriers (10 μL) were applied to a TEM grid (Cu 200 mesh covered with a collodion membrane, Nisshin EM, Japan) for 1 min and dried with a filter paper. The peptide-mounted TEM grid was blocked with TBST (20 mM Tris-HCl buffer, 150 mM NaCl, 0.05% Tween20, pH 7.4) containing 0.1% BSA (Sigma) for 15 min, washed with TBST (twice), reacted with gold colloidal particle (10 nm)-labeled goat anti-biotin antibody (10 μg mL^{-1}) in TBST containing 1% BSA at 25 °C for 30 min, washed with TBST (3 times) and water (once), and negatively stained in the same manner described above for the measurements.

Atomic Force Microscopy (AFM). A silicon wafer [p-Si(100), Mitsubishi Materials Co., Japan] was cut into 7 × 7-mm pieces and subjected to ultrasonic cleaning in acetone for 5 min (3 times), followed by photocleaning with an ultraviolet ozonizer (Kenix KUV-100, Kenix, Japan) at room temperature for 10 min. Droplets of the solution containing self-assembled

peptide nanocarriers (10 μL) were applied to the silicon substrates for 1 min, washed with water, and dried with a filter paper. All AFM samples were dried *in vacuo* before AFM measurements were conducted (MFP-3D-SA-J, Asylum Technologies, Japan) with a cantilever (Olympus OMCL-AC240TS-C3, Olympus, Japan).

Cell Culture. Human cervical carcinoma (HeLa) cells were cultured in Dulbecco's modified Eagle's medium (DMEM) supplemented with 10% fetal bovine serum and 100 U mL^{-1} penicillin and 100 μg mL^{-1} streptomycin (Medium 1). HeLa cells were seeded with 5.0×10^4 cells in glass-bottomed culture chambers with 4 compartments (35 mm, CELLview, Greiner Bio-one, Japan). After 1-day culture, the cellular delivery assay was conducted at 37 °C and 5% CO_2 atmosphere in an incubator SCA-165 DRS (Astec, Japan).

Confocal Laser Scanning Microscopy. After removal of the medium from seeded cell samples, the cells were gently washed three times with Medium 1. The cells were incubated with an equivolume mixture of DMEM (50 μL) and 6 h-matured-solution containing self-assembled peptide nanocarriers in 20 mM phosphate buffer, 150 mM NaCl, pH 7.2 (50 μL) at 37 °C and 5% CO_2 atmosphere for 4 h. The cells were washed twice with 1xHEPES (10xHEPES = 200 mM HEPES, 1150 mM NaCl, 54 mM KCl, 8 mM $MgCl_2$, 138 mM D-glucose, pH 7.4), and then with 0.4% trypan blue solution (Sigma), followed by staining nuclei with Hoechst33342 (1 mM, 1 μL) in 1xHEPES (100 μL) for 10 min and washing with 1xHEPES twice. Cells were analyzed in 1xHEPES with a confocal laser scanning microscopy (CLSM) LSM 700 (Carl Zeiss, Japan) equipped with ZEN2009 software (Carl Zeiss) for acquisition of fluorescence images. The TMR and Hoechst33342 were excited with the 555 and 405 nm lasers and fluorescence signals were recorded using filters of 580 and 440 nm, respectively.

Doxorubicin Encapsulation. The peptide stock solution in TFE was transferred into a microtube, dried with a N_2 gas stream, then dried *in vacuo* for 1 h. Solvent (20 mM phosphate buffer, 150 mM NaCl, pH 7.2) was added to the microtube and the obtained aqueous solution was sonicated with a Kaijo Sono Cleaner (CA-44800, Japan) at 50 °C for 2 min to allow peptides being in monomer/oligomer states, then filtered through a spin filter (Ultrafree-MC-VV, 0.1 μm, Millipore). The filtrate was mixed with Dox-containing aqueous solution in an equivolume ratio and incubated in a low temperature incubator (Fukushima

FMU-0531, Japan) at 25 °C for 6 h for Dox encapsulation. The obtained samples were served for cell viability measurements.

For quantification of the amounts of Dox encapsulated in the peptide self-assemblies, the obtained samples were further filtered through ultrafiltration membrane filters (Amicon Ultra-0.5 mL Ultracel-3K (COMW 3 kDa), Amicon Ultra-0.5 mL Ultracel-10K (COMW 10 kDa) or Amicon Ultra-0.5 mL Ultracel-100K (COMW 100 kDa), Millipore). Fluorescence spectra for the filtrates were acquired on a Hitachi F4500 fluorescence spectrophotometer (λ_{ex} = 480 nm, λ_{em} = 500–800 nm). Amounts of peptides passed through the membrane filter were also determined by Hitachi LaChrom Elite HPLC system (Japan) using Cosmosil $5C_{18}$-AR-II packed columns (4.6 x 150 mm, Nacalai Tesque, Japan) with a linear gradient of acetonitrile/0.1% TFA at a flow rate of 1.0 mL min^{-1} (acetonitrile content: 0 % (t = 0 min) → 100 % (t = 30 min); UV detection: 220 nm).

For gel filtration chromatography of Dox only and Dox-loaded, samples ([Dox] = 10 μM and [RU006] = 100 μM in 20 mM sodium phosphate buffer, 150 mM NaCl, pH 7.2 at 25 °C with 6-h incubation) were analyzed by Hitachi LaChrom Elite HPLC system (Japan) using Cosmosil 5Diol-1000-II (7.5 x 300 mm, Nacalai Tesque, Japan) with an isocratic flow of 20 mM sodium phosphate buffer, 150 mM NaCl, pH 7.2 at flow rate of 1.0 mL min^{-1} at 220 nm detection.

Cell Viability Measurements. HeLa cells were seeded with 1.0×10^4 cells in a 96-well plate and cultured at 37 °C and 5% CO_2 atmosphere for 24 h. An equivolume mixture of DMEM (100 μL) and 6 h-matured-solution containing self-assembled peptide nanocarriers with/without Dox in 20 mM phosphate buffer, 150 mM NaCl, pH 7.2 (100 μL) was prepared and subsequently transferred into the 96-well plate, and incubated at 37 °C and 5% CO_2 atmosphere for 24 h. Cells were washed with 1xHEPES twice, followed by treated with 1xHEPES (50 μL) and 0.2% Cell Counting-kit 8 (50 μL, Dojindo Molecular Technologies, Japan) at 37 °C and 5% CO_2 atmosphere for 30 min for cell viability measurements.

(2) Characterization data for the peptides.

(2-1) Characterization of NLS-RU006.

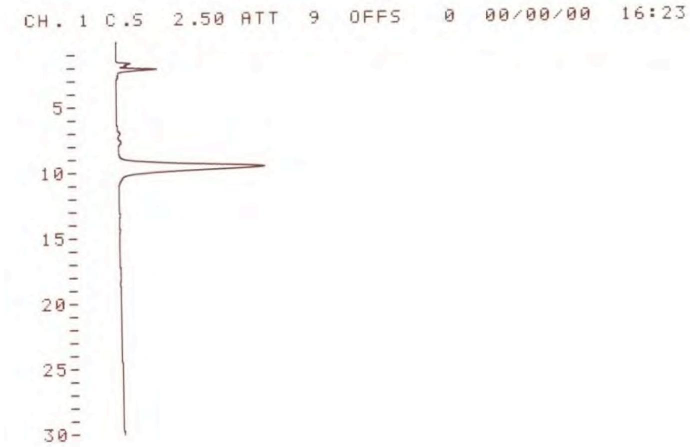

Figure S1. HPLC profile for NLS-RU006. Conditions: acetonitrile content: 23 % (t = 0 min) → 26 % (t = 30 min); UV Detection: 220 nm.

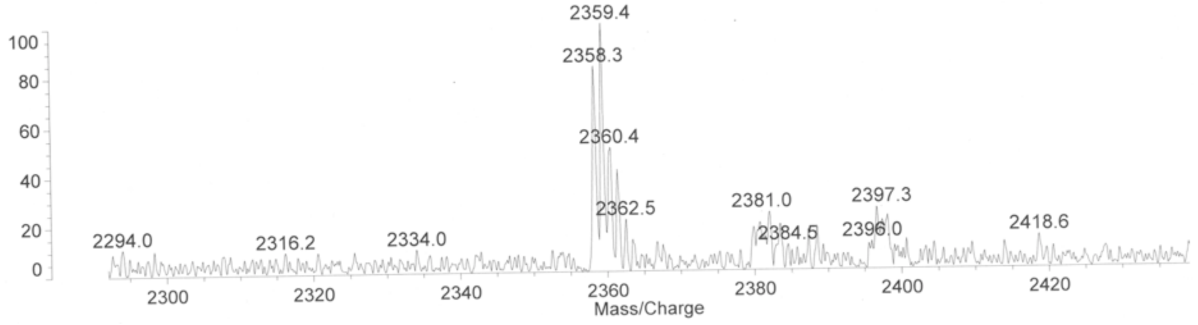

Figure S2. HPLC profile for NLS-RU006. Calculated molecular weight: 2357.9; observed molecular weight 2358.8 $[M + H]^+$, 2381.0 $[M + Na]^+$, 2396.0 $[M + K]^+$.

(2-2) Characterization of R8-RU006.

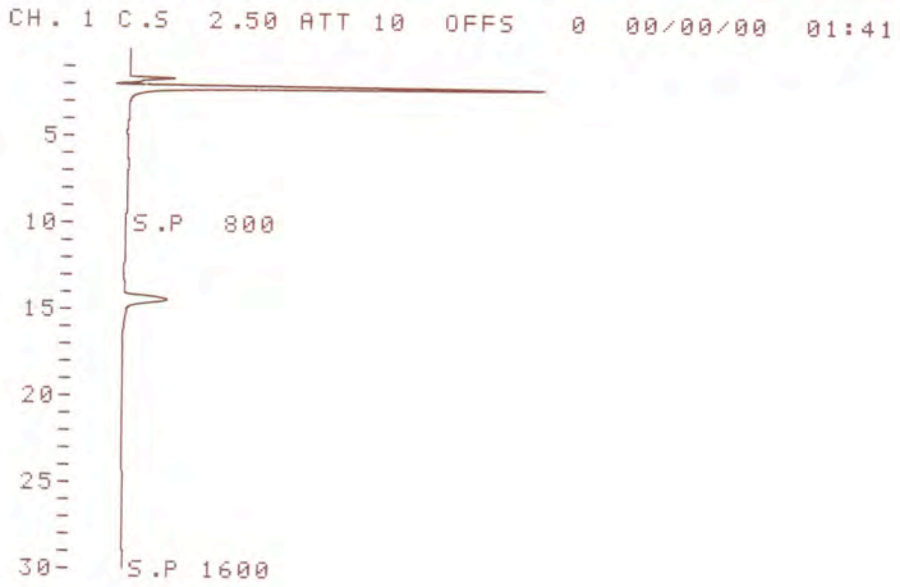

Figure S3. HPLC profile for R8-RU006. Conditions: acetonitrile content: 19 % (t = 0 min) → 33 % (t = 30 min); UV Detection: 220 nm.

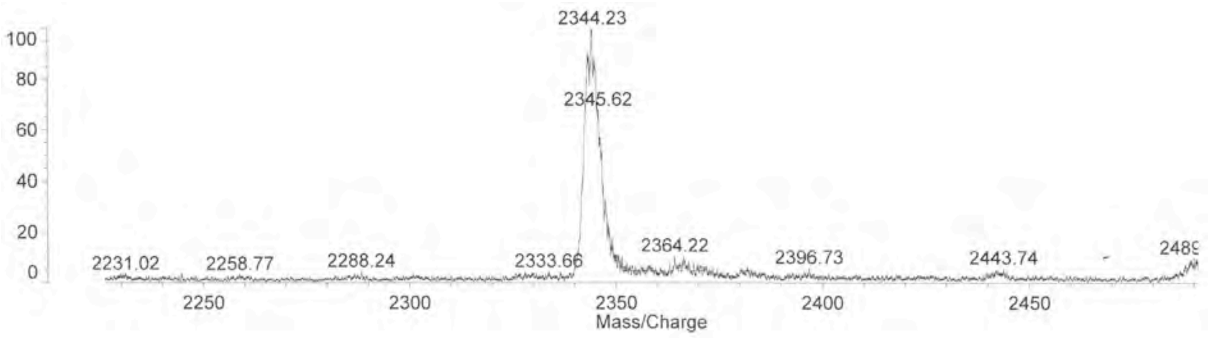

Figure S4. HPLC profile for R8-RU006. Calculated molecular weight: 2344.8; observed molecular weight 2344.2 $[M + H]^+$.

(2-3) Characterization of Tat-RU006.

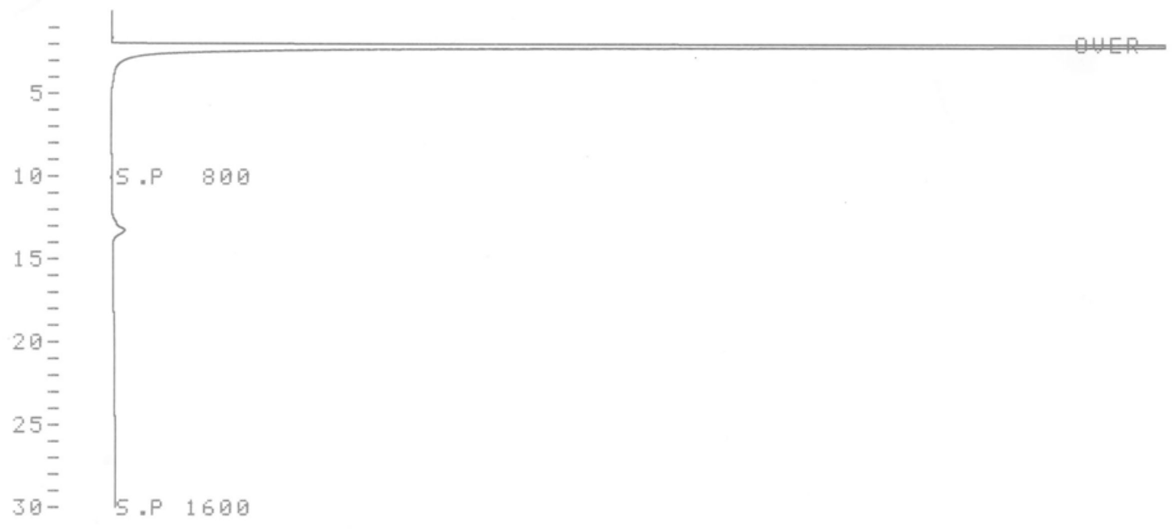

Figure S5. HPLC profile for Tat-RU006. Conditions: acetonitrile content: 22 % (t = 0 min) → 30 % (t = 30 min); UV Detection: 220 nm.

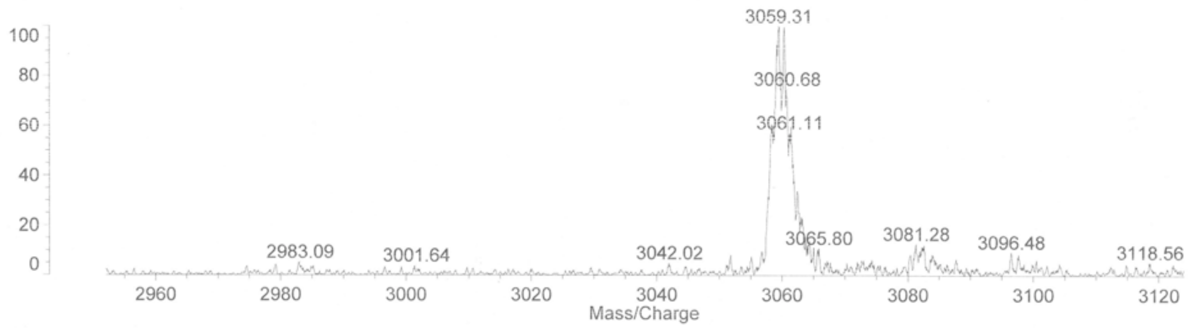

Figure S6. HPLC profile for Tat-RU006. Calculated molecular weight: 3058.6; observed molecular weight 3059.3 $[M + H]^+$.

(2-4) Characterization of TMR-RU006.

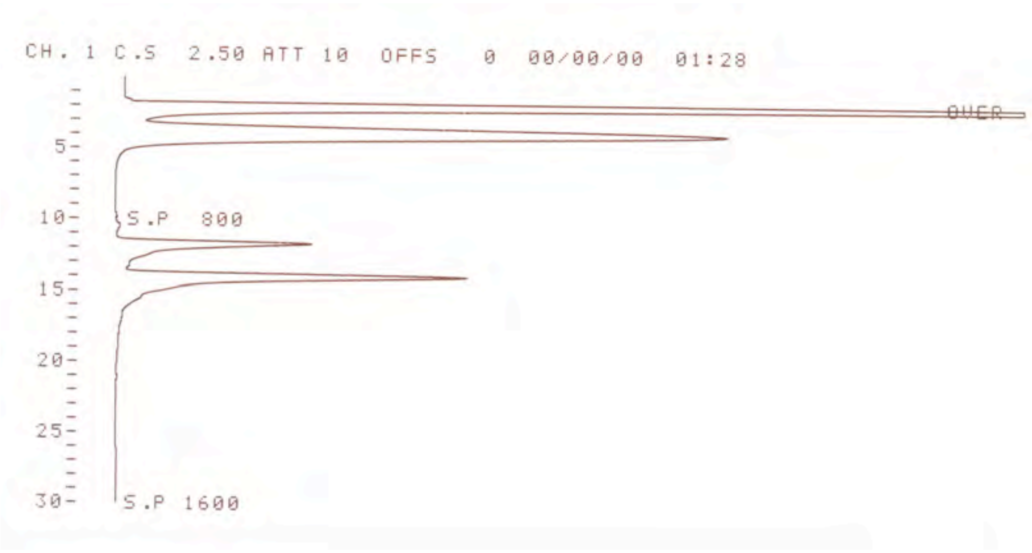

Figure S7. HPLC profile for TMR-RU006. Two peaks for 5- and 6-isomers (at 12-14 min) were observed in the chromatogram. Conditions: acetonitrile content: 30 % (t = 0 min) → 40 % (t = 30 min); UV Detection: 220 nm.

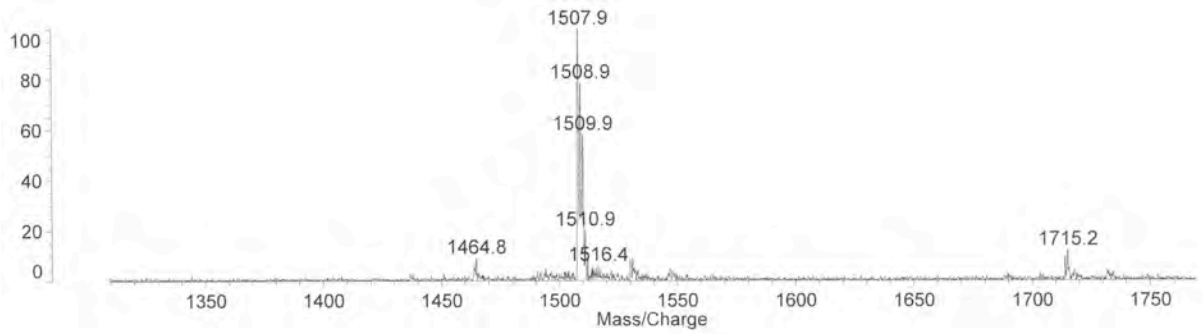

Figure S8. HPLC profile for TMR-RU006. Calculated molecular weight: 1507.8; observed molecular weight 1507.9 $[M + H]^+$.

(3) **Characterization of the Self-Assembled Peptide Nanocarriers of Entry 1.**

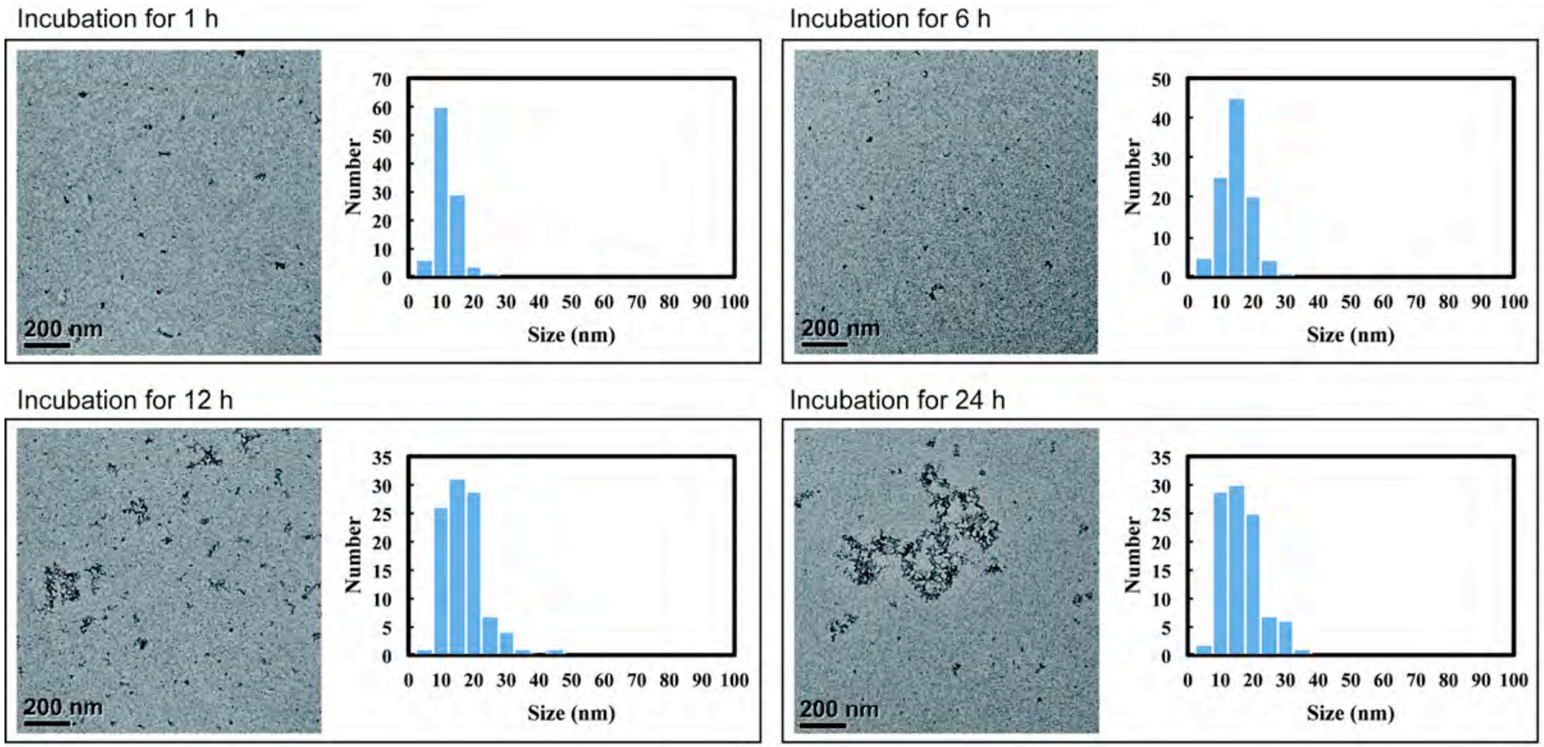

Figure S9. TEM images of the self-assembled peptide nanocarriers of Entry 1 as a function of elapsed time ([peptide] = 100 μM in 20 mM phosphate buffer, 150 mM NaCl, pH 7.2 at 25 °C). Samples were stained with 2% phosphotungstic acid.

(4) Characterization of the Self-Assembled Peptide Nanocarriers of Entries 1, 2, 3 and 4.

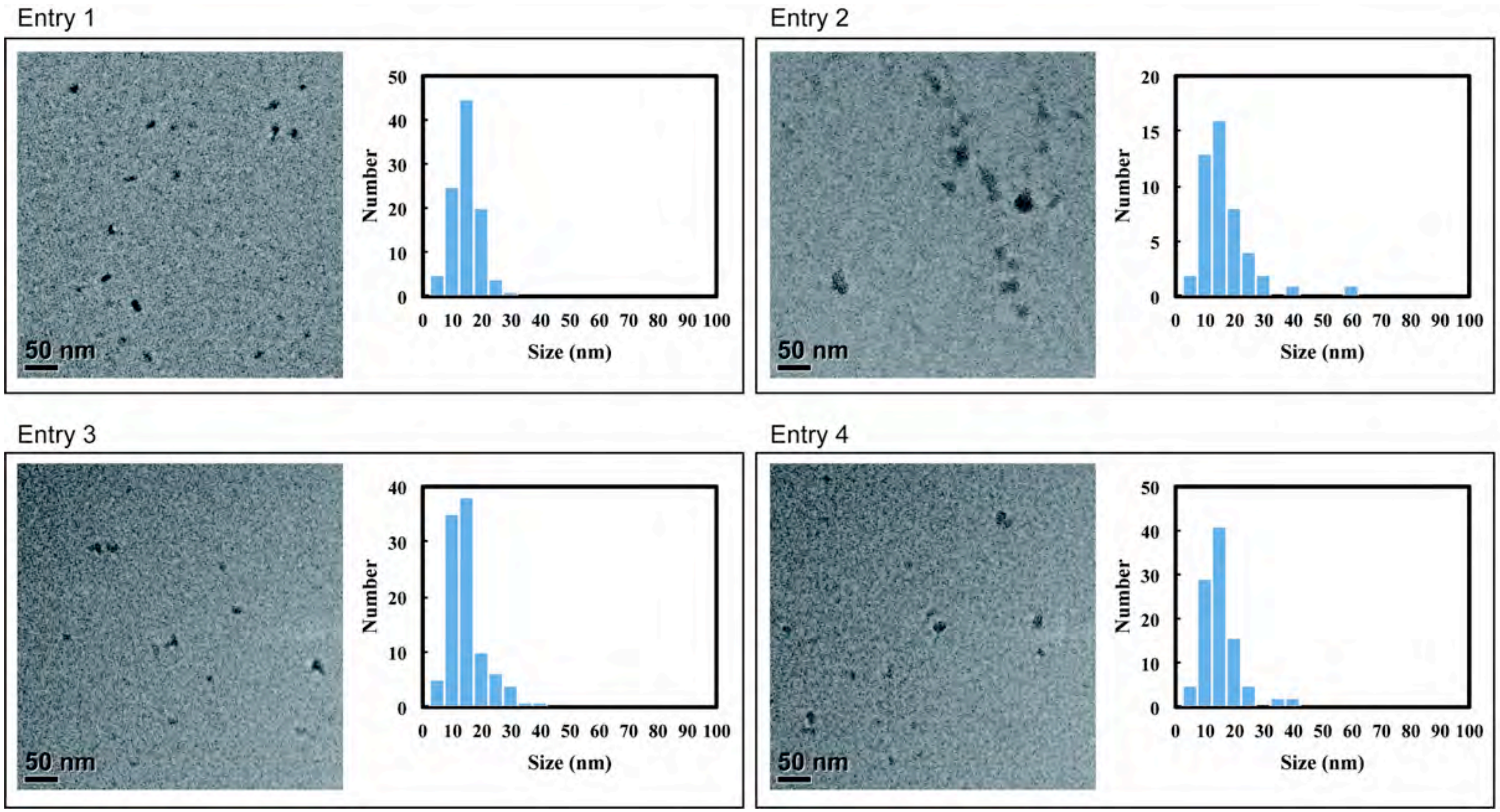

Figure S10. TEM images of the self-assembled peptide nanocarriers of Entries 1, 2, 3, and 4 at 6 h after self-assembly started ([peptide] = 100 μM in 20 mM phosphate buffer, 150 mM NaCl, pH 7.2 at 25 °C). Samples were stained with 2% phosphotungstic acid.

(5) Cytotoxicity of Self-Assembled Peptide Nanocarriers for HeLa cells.

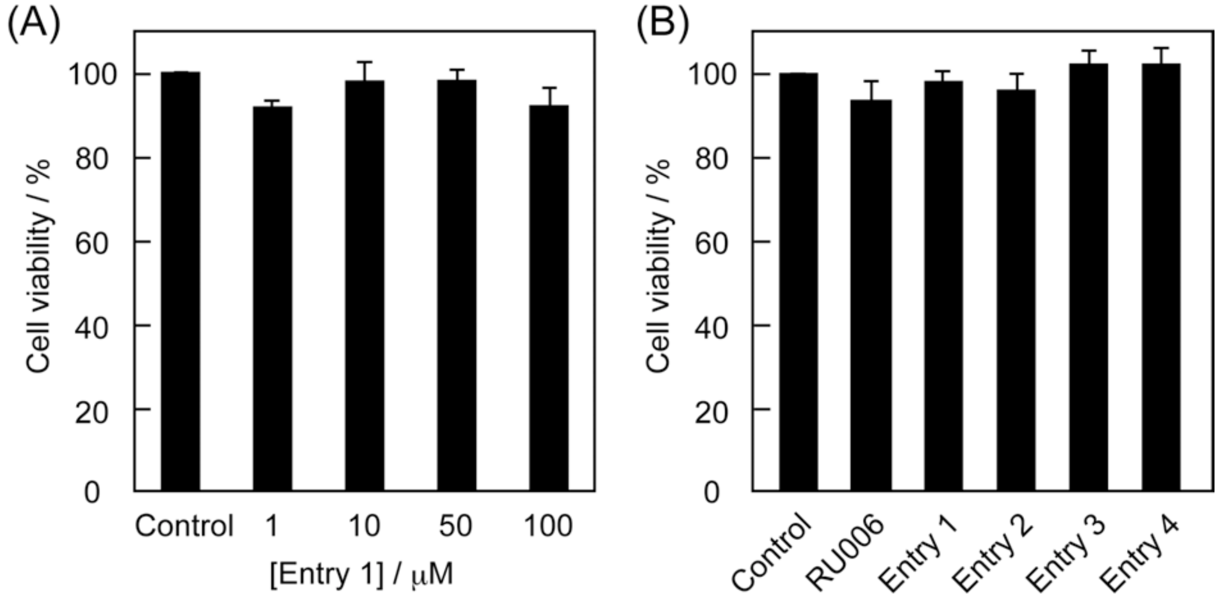

Figure S11. Cytotoxicity of self-assembled peptide nanocarriers for HeLa cells at 37 °C and 5% CO_2 atmosphere for 24 h depending on (A) concentration of the peptides of Entry 1 ([peptide] = 1, 10, 50, and 100 μM) and (B) differences in amino acid sequences of CPP/NLS moieties ([peptide] = 50 μM) (mean ± SD, n ≥ 3). Control experiments involve cell culture without peptides.

(6) Gel filtration chromatography of Dox only and Dox-loaded RU006 Peptide Nanocarrier.

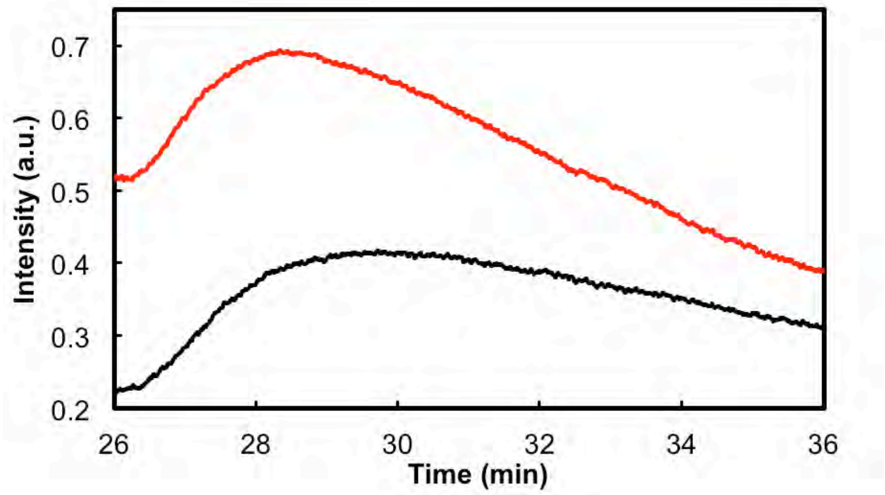

Figure S12. Comparison of Dox peaks in gel filtration chromatograms for Dox only (black line) and Dox-loaded self-assembled RU006 peptide nanocarriers (red line) (sample preparation: [Dox] = 10 μM and [RU006] = 100 μM in 20 mM sodium phosphate buffer, 150 mM NaCl, pH 7.2 at 25 °C for 6 h).

(7) Correlation between fluorescence intensity and concentration of Dox.

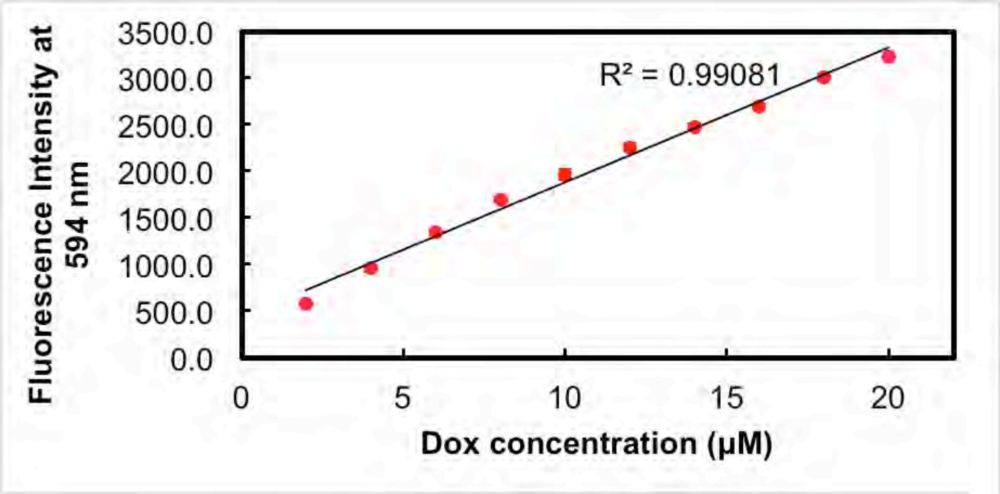

Figure S13. Correlation between fluorescence intensity from Dox at 594 nm (λ_{ex} = 480 nm) and Dox concentration in 20 mM sodium phosphate, 150 mM NaCl, pH 7.2 at 25 °C.

(8) Doxorubicin Encapsulation by RU006 Peptide Nanocarrier Characterized by Ultrafiltration Assay.

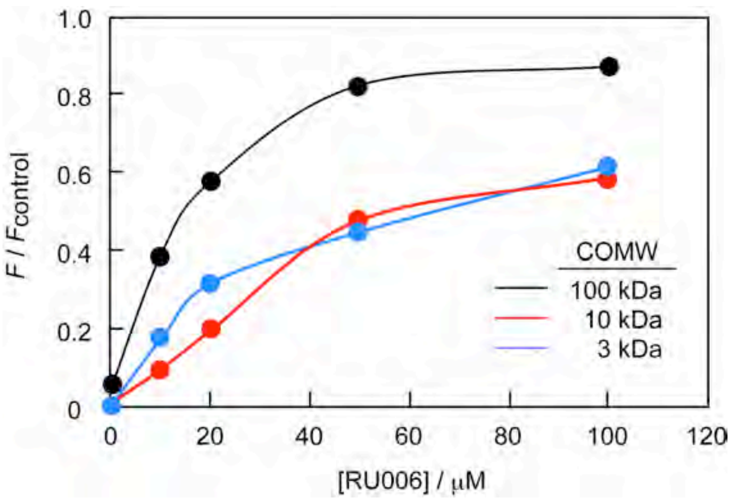

Figure S14. Relative fluorescence intensity from Dox-loaded self-assembled RU006 peptide nanocarrier at 594 nm (λ_{ex} = 480 nm) after ultrafiltration through ultrafiltration membrane filters (Amicon Ultra-0.5 mL Ultracel-3K, Amicon Ultra-0.5 mL Ultracel-10K, and Amicon Ultra-0.5 mL Ultracel-100K) as a function of RU006 concentration (sample preparation: [Dox] = 10 μM and [RU006] = 0, 10, 20, 50, and 100 μM in 20 mM sodium phosphate buffer, 150 mM NaCl, pH 7.2 at 25 °C for 6 h). $F_{control}$ indicates Dox fluorescence intensity without peptides before ultrafiltration.

(9) Doxorubicin Encapsulation by RU006 Peptide Nanocarrier Characterized by HPLC.

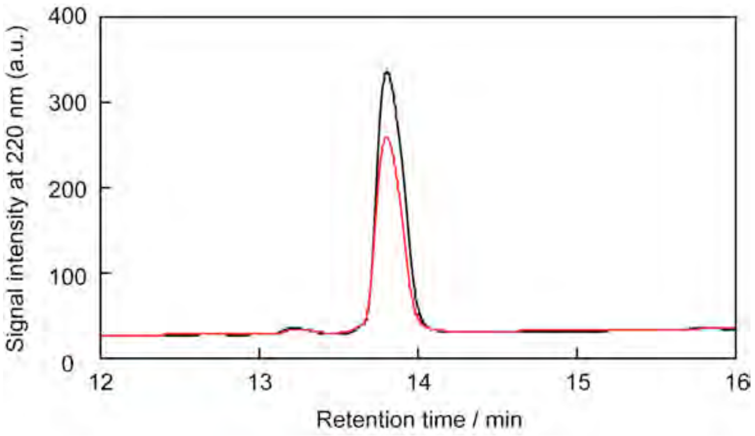

Figure S15. HPLC profiles of Dox-loaded self-assembled RU006 peptide nanocarriers before (black line) and after (red line) ultrafiltration through a membrane filter of Amicon Ultra-0.5 mL Ultracel-100K (COMW 100 kDa) (sample preparation: [Dox] = 10 μM and [RU006] = 100 μM in 20 mM sodium phosphate buffer, 150 mM NaCl, pH 7.2 at 25 °C for 6 h).

(10) Doxorubicin Release from RU006 Peptide Nanocarrier Characterized by Ultrafiltration Assay.

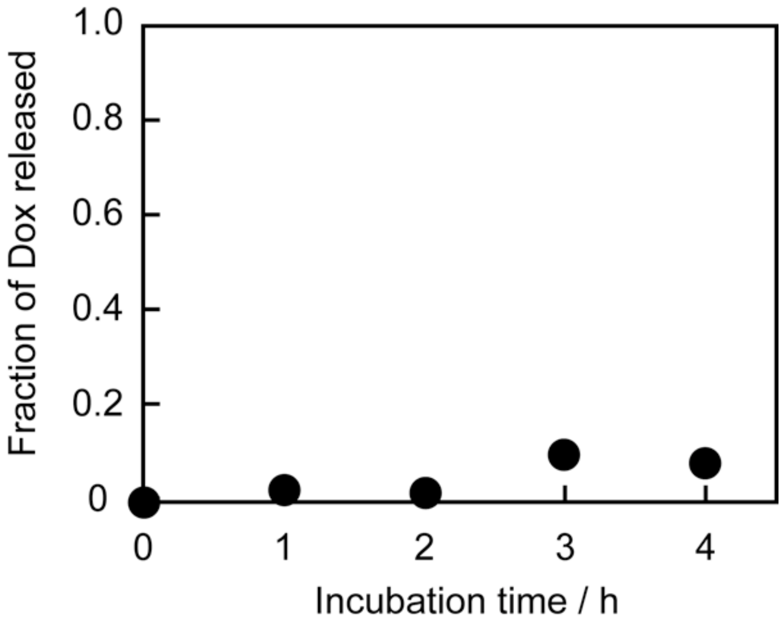

Figure S16. Fraction of Dox released from Dox-loaded self-assembled RU006 peptide nanocarrier as a function of time. Sample preparation: Dox (10 μM) and RU006 (100 μM) were incubated in 20 mM sodium phosphate buffer, 150 mM NaCl, pH 7.2 at 25 °C for 6 h for Dox encapsulation. The matured solution was mixed with cell culture medium in an equivolume ratio and divided into five portions to prepare portions 1-5. The portion 1 was subsequently filtered through an ultrafiltration membrane filter (Amicon Ultra-0.5 mL Ultracel-100K) and monitored by fluorescence spectroscopy (λ_{ex} = 480 nm). Similarly, the portions 2, 3, 4, and 5 were incubated at 37°C for 1, 2, 3, and 4 h, respectively.

(11) References.

S1 Tomizaki, K.-Y.; Wakizaka, S.; Yamaguchi, Y.; Kobayashi, A.; Imai, T. *Langmuir* **2014**, *30*, 846–856.

S2 Chan, W. C.; White, P. D. *Fmoc Solid Phase Peptide Synthesis: A Practical Approach*, Oxford University Press, New York, 2000, pp. 41–76.

S3 Sadqi, M.; Lapidus, L. J.; Muñoz, V. *Proc. Natl. Acad. Sci. USA*, **2003**, *100*, 12117–12122.
